# Recombinant hemagglutinin displaying on yeast reshapes congenital lymphocyte subsets to prompt optimized systemic immune protection against avian influenza infection

**DOI:** 10.3389/fmicb.2023.1153922

**Published:** 2023-05-31

**Authors:** Han Zhang, Zexing Li, Huixia Zhang, Yanyu Guo, Xinyi Zhang, Lilin Zhang, Liu Yang, Shujun Li, Changyan Li, Daqing Cui, Ruyu Xie, Yongqing Li, Jinhai Huang

**Affiliations:** ^1^School of Life Sciences, Tianjin University, Tianjin, China; ^2^Institute of Animal Husbandry and Veterinary Medicine, Beijing Academy of Agricultural and Forestry Sciences, Beijing, China

**Keywords:** avian influenza virus, *Saccharomyces cerevisiae*, innate immunity, vaccine, chicken

## Abstract

**Introduction:**

Prophylactic vaccination is regarded as the most effective means to control avian flu infection. Currently, there is a need for a universal vaccine that provides broad and long-lasting protection against influenza virus. Meanwhile, although yeast-based vaccines have been used in clinic, studies are still required to further understand the molecular mechanism of yeast-based vaccines under physiological conditions.

**Methods:**

We generated a yeast-based vaccine against influenza hemagglutinin (HA) of H5, H7 and H9 using surface displaying technology and evaluated the protective efficacy of chickens after exposure to H9N2 influenza virus.

**Results:**

Oral yeast vaccine provided less clinical syndrome, reduced viral loading and alleviated airway damage significantly. Compared to the commercial inactivated vaccine, yeast vaccine stimulated the activation of splenic NK and APCs cells and boosted TLR7-IRF7-IFN signaling in spleen. Meanwhile, γδ T cells in the bursa of Fabricius were activated and the innate lymphoid cells (ILCs) in the bursa of Fabricius promoted the CILPs to differentiate to ILC3 cells in oral yeast birds. Moreover, the reshaped gut microbiota and a suppressed Th17-IL17-mediated inflammation in intestine was observed in oral yeast chickens, which might facilitate the recovery of intestinal mucosal immunity upon virus infection. Collectively, our findings suggest that oral yeast based multivalent bird flu vaccines provide an attractive strategy to update host defense function via reshapes of multi-systemic immune homeostasis.

## Introduction

Avian influenza virus (AIV) is an acute and highly contagious zoonotic pathogen that poses a serious threat to public health and the poultry industry. Avian influenza virus subtypes H5N8 and H7N9 are highly pathogenic avian influenza (HPAI) and can cause severe mortality, while H9N2 is a low pathogenic avian influenza (LPAI), which does the same. The first epidemic of HPAI occurred in Guangxi province and quickly spread to 16 provinces of China in 2004, and it resulted in great economic losses and was accompanied by a high mortality rate from human influenza in China (Swayne et al., 2014; Chen et al., 2018). Vaccination is considered an efficient strategy to control AIV infection, and massive vaccination programs were launched to fight against bird flu infection since then. Usually, combined H5N8, H7N9, and H9N2 inactivated vaccines were widely used in poultry (Allen et al., 2019; Christensen et al., 2019). However, the commercial inactivated influenza vaccines offer little protection against pandemic influenza virus strains (Bajic et al., 2019). Meanwhile, the vaccination procedures of inactivated vaccines were time-consuming and relied on hand labor.

Hemagglutinin protein (HA) of influenza A viruses, a homotrimeric glycoprotein in architecture, is the only antigen present on the viral surface and contains several glycosylation sites (Bangaru et al., 2019; Vahey and Fletcher, 2019). Crystallographic studies have shown that HA forms a trimer embedded on the viral envelope surface, and each monomer consists of a globular head (HA1) and a rod-like stalk region (HA2). Moreover, the latter HA2 region is more conserved among different HA subtypes, and it is considered the primary target for universal vaccines (Xuan et al., 2011). Recombinant HA protein subunit vaccines were evaluated for their safety and efficacy in humans and chickens in previous studies (Wu et al., 2010; Kim et al., 2017; Lei et al., 2020). Several universal influenza vaccines including a limited number of antigens that have epitopes that are conserved across different influenza virus subtypes are in development to provide protection against diverse influenza virus subtypes (Arevalo et al., 2022). Continued efforts to further characterize the phenotype and function of these vaccines will guide the development of more effective vaccines, which provide long-lasting protective efficacy against both seasonal and pandemic influenza viruses (Cho and Wrammert, 2016).

Recently, gut microbiota (GM) modulation approaches are considered one of the most promising strategies to improve animal health and welfare in commercial poultry production. The immune system of avian species, such as chickens, is thought to be very different from mammalian species (Kaiser, 2010). Unique features, such as cecum tonsils (the largest lymphoid aggregates in the bird's gut) and the bursa of *Fabricius* (a B-cell powerhouse), both of which are located in the distal region of the intestinal mucosa, make the avian gut-associated immune system a very specific anatomical and immunological landscape compared to that of mammals. Therefore, modulation of the chicken gut microbiota might provide a stronger positive effect on chicken health, and there is a great need to understand the influence of GM modulation on the chicken gut-associated immune system.

Yeast is considered to be a simple and cost-effective protein expression host, and yeast-based vaccines have gained popularity due to their rapidly engineered and manipulated characteristics to express foreign antigens and viral epitopes (Kiflmariam et al., 2013; Jiang et al., 2014; Guan et al., 2015; Sen and Mansell, 2020). A yeast displaying influenza H7N9 oral vaccine provides protection against the lethal H7N9 virus challenge in mice (Lei et al., 2020). Recent studies demonstrated that oral recombinant *Saccharomyces cerevisiae* successfully introduced recombinant protein and DNA into rabbit dendritic cells (Franzusoff et al., 2005). Meanwhile, yeast is preferred in GM modulation approaches because of its multiple effects as a probiotic, including anti-toxin, resistance to intestinal pathogens, and maintenance of the integrity of the intestinal epithelial mucosa. However, the influence of yeast-based vaccines on gut-associated immune systems and GM modulation of chickens were not fully understood.

In this study, we try to present an alternative strategy for inducing universal immunity against distinct influenza virus strains. Instead of focusing on immunogens to elicit antibodies against epitopes that are conserved among many different influenza virus strains, we designed a novel trivalent recombinant protein yeast surface display-based vaccine, which simultaneously expressed the HA regions of H5N8, H7N9, and H9N2 subtypes and lineages. The immunogenicity and protective efficacy of this vaccine were tested by using the chicken model, and our experimental results demonstrated that the yeast vaccine is safe, efficient, and inexpensive for commercial production. Further studies revealed that our triple yeast vaccine can selectively activate immune organs (bursa of *Fabricius* and spleen), suppress parenchymal organ inflammatory responses, promote tissue-resident ILC3 cell proliferation, exert protective functions in bursa, and lead to differentiation of ILCs, NKs, and γδT cells in different tissues, all of which collectively contributed to the defense response against influenza virus infection of vaccinated chickens.

## Results

### Construction of a trivalent recombinant *S. cerevisiae* strain expressing HA proteins of subtypes H5N8, H7N9, and H9N2

**Figure 1:**
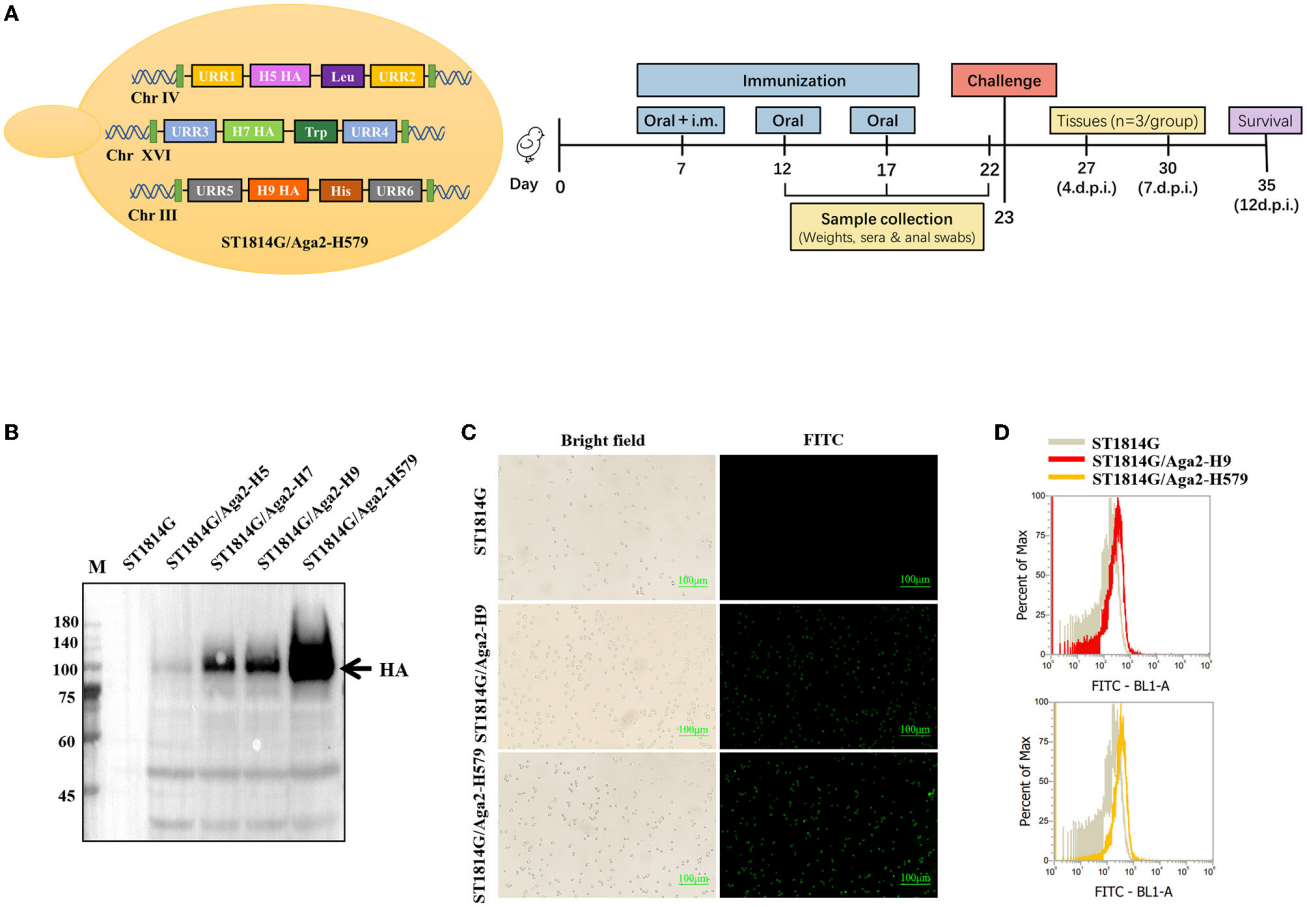
Analysis of HA protein expression in recombinant Saccharomyces cerevisiae strains. **(A)** Schematic diagram of yeast construction (Left) and animal evaluation experiments (right). **(B)** The Western blot analysis of HA expression in recombinant S. cerevisiae. Lane M. Blue Plus IV Protein Maker (10–180 kDa); Lane 1. ST1814G; Lane 2. ST1814G/Aga2-H5; Lane 3. ST1814G/Aga2-H7; Lane 4. ST1814G/Aga2-H9; Lane 5. ST1814G/Aga2-H579. **(C)** ST1814G/Aga2-H9 cells and ST1814G/Aga2-H579 cells were incubated with mouse anti-His-tag antibody followed by fluorescein isothiocyanate (FITC)-conjugated anti-mouse IgG. Treated yeast cells were fixed on glass slides, and the localization of HA was analyzed by fluorescence microscope. **(D)** The expression profile of recombinant protein was also determined by flow cytometry. The gray lines (dark shades) represent ST1814G cells, the red line stands for ST1814G/Aga2-H9 cells, and the yellow line stands for ST1814G/Aga2-H579 cells.

To meet the requirements of developing safe and effective influenza oral vaccines, we developed a trivalent recombinant HA protein vaccine by yeast surface display manner using *Saccharomyces Cerevisiae* strain (MATa aga1 his3Δ200 leu2Δ0 lys2Δ0 trp1Δ63 ura3Δ0 met15Δ0) as shown in Figure 1A. The positive transformed yeast clones were further confirmed by analyzing yeast genomic DNA for correct insertion. The recombinant *S. Cerevisiae* strain expressing H9/HA was named ST1814G/Aga2-H9, and the recombinant *S. Cerevisiae* strains expressing HA proteins of H5N8, H7N9, and H9N2 were named ST1814G/Aga2-H579 in this study. The Western blot results indicated that the HA proteins of H5N8, H7N9, and H9N2 strains were all expressed successfully in our engineered yeast strains (Figure 1B). Immunofluorescence assay and flow cytometry assay demonstrated that HA proteins from both ST1814G/Aga2-H9 and ST1814G/Aga2-H579 were expressed on the yeast surface successfully (Figures 1C, D). Overall, these results indicate that the trivalent HA proteins were successfully expressed and located on the cell surface of our engineered yeast vaccine cells.

### Effects of oral yeast vaccines on body weight, organ indices, specific antibody production, and virus challenge of chickens

**Figure 2:**
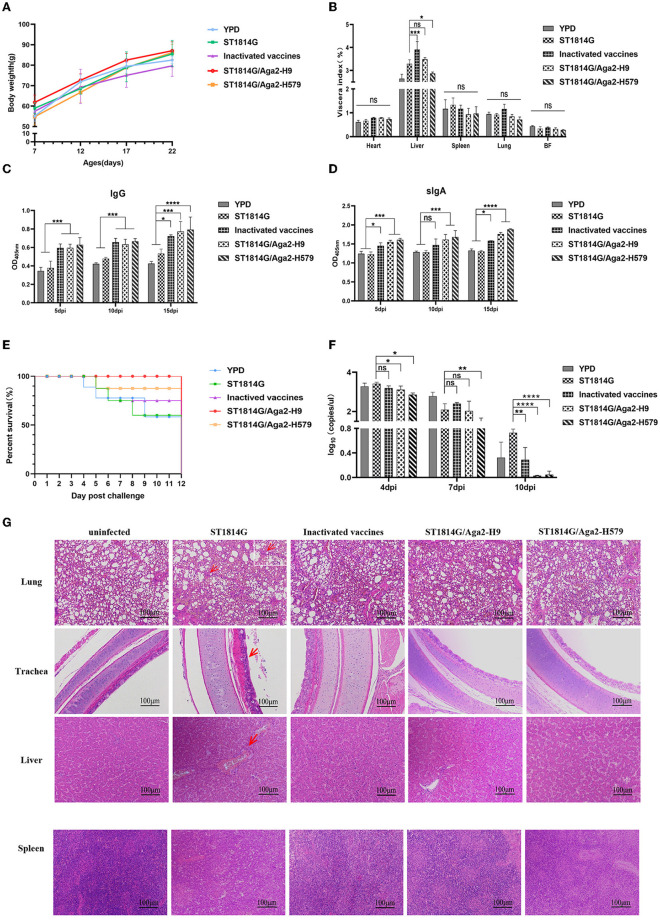
Effects of oral recombinant yeast vaccine on growth, viscera index, special antibody production, survival curve, virus titer, and histopathological sections after viral challenge. **(A)** The weight of chickens was measured at 5 days intervals, and three chickens were randomly selected from each group to graph the change in weight. **(B)** 5 days after the third immunization, the organ index (wet weight of viscera/body weight) for each chicken in each group was recorded, including the heart, liver, spleen (×10), lungs, and bursa of *Fabricius*. **(C, D)** The secretion levels of H9N2 HA-specific IgG in serum and sIgA in anal swabs of vaccinated chickens were determined by ELISA, and the differences were compared among groups, respectively. **(E)** The survival curve was recorded after the H9N2 virus was injected into seven chickens in each group, and the percentage of survival was calculated. **(F)** Virus titers in blood were measured by absolute qPCR assay on days 4, 7, and 10 after virus infection (dpi: day post-infection). **(G)** Microscopic lesions of tracheal epithelial cells, alveolar cells and hepatocytes, and spleen cells in experimentally infected birds were demonstrated by hematoxylin and eosin staining (HE). Left to right: uninfected group; ST1814G group, inactivated vaccine group, ST1814G/Aga2-H9 group, and ST1814G/Aga2-H579 group after being infected. The data in the figures were obtained from three independent experiments and represent the averages ± SD. The significance of differences was determined by a two-way analysis of variance (*p < 0.05; **p < 0.01; ***p < 0.001; ****p < 0.0001 or ns, no significance). The red arrow indicates massive infiltration of lymphocytes in tissues.

To monitor the safety of our engineered yeast vaccines, the weight of the whole body and different organs was recorded during the three immunization procedures. As shown in Figures 2A, B, oral-engineered yeast vaccines had no negative effect on the growth of the chickens but rather tend to mildly increase the weight of chickens. The organ index results showed a considerable disturbance of the liver by the inactivated vaccine, which may trigger a strong inflammatory response or macrophage infiltration that leads to relative liver enlargement. In contrast, the oral yeast vaccine group, including ST1814G/Aga2-H9 and ST1814G/Aga2-H579, did not cause liver enlargement, suggesting that the oral yeast vaccine could reduce the potential for further damage to the organism.

To detect the special antibodies production of HA protein by ST1814G/Aga2-H9 and ST1814G/Aga2-H579, the levels of IgG in sera and IgA in anal swabs were determined by the ELISA assay (Figures 2C, D). The levels of specific IgG and IgA were significantly higher in oral yeast vaccine and inactivated vaccine groups than those in the ST1814G or YPD groups as early as 5 days after the first immunization, and the specific antibody levels were increased progressively with the immunization times. The results indicated that, in comparison with the control group, both the oral yeast vaccine and the traditional inactivated vaccine were able to stimulate a strong humoral immune response against the antigen HA protein in chickens.

At 5 days after the third immunization, the chickens were challenged with H9N2 (A/Hebei/218/2010), and the survival rate was recorded as in Figure 2E. Only 53% of chickens survived in the YPD and ST1814G groups, and 71% of chickens survived in the inactivated vaccine group; the ST1814G/Aga2-H579 group had a high survival rate of 86%, and the ST1814G/Aga2-H9 group was able to provide 100% protection for the chickens. It indicated that oral administration of ST1814G/Aga2-H9 yeast or ST1814G/Aga2-H579 yeast provided better protection for chickens after H9N2 virus infection. Consistent with the survival rate experiments, virus titer experiments, which were measured in the blood of each group at 4, 7, and 10 days after the H9N2 infection, also indicated that oral administration of ST1814G/Aga2-H9 yeast or ST1814G/Aga2-H579 yeast could additionally decrease the virus titer of chickens (Figure 2F).

We further checked the histopathological lesions of each group (Figure 2G). Compared to the group without virus infection, the ST1814G group showed significantly higher airway lesions and pneumonia symptoms that are characterized by interstitial lung congestion with hemorrhage and massive infiltration of lymphocytes, macrophages, and granulocytes. In contrast, the lung tissue of the other three immunization groups had only scattered lymphocyte infiltrates. Meanwhile, the main liver lesions were characterized by intrahepatic erythrocytosis with lymphocytic infiltration around the vessels in the ST1814G group, whereas the immunized groups showed only scattered lymphocytic infiltration or no symptom in the liver tissue. Spleen histology showed blurred boundaries between the red and white marrow in the ST1814G group and the inactivated vaccine group, suggesting that an inflammatory response occurred in the spleen, whereas the red and white marrow were more clearly defined in the oral yeast immunization groups. Taken together, these results showed that the oral yeast vaccines were effective in defending against and clearing viruses and hence protecting immunized chickens from deadly injuries.

### Oral yeast vaccine regulates tissue-resident innate immune cell abundance to protect chickens from lethal viral damage

Previous studies reported that the oral yeast vaccination was able to activate innate immune cells, such as macrophage and NK cells (Jensen et al., 2007; Liu et al., 2021; Walachowski et al., 2022). To test whether our engineered yeast vaccines could influence innate immune cell abundance, we determined the expression level of molecule markers of important innate immune cells in different groups.

**Figure 3:**
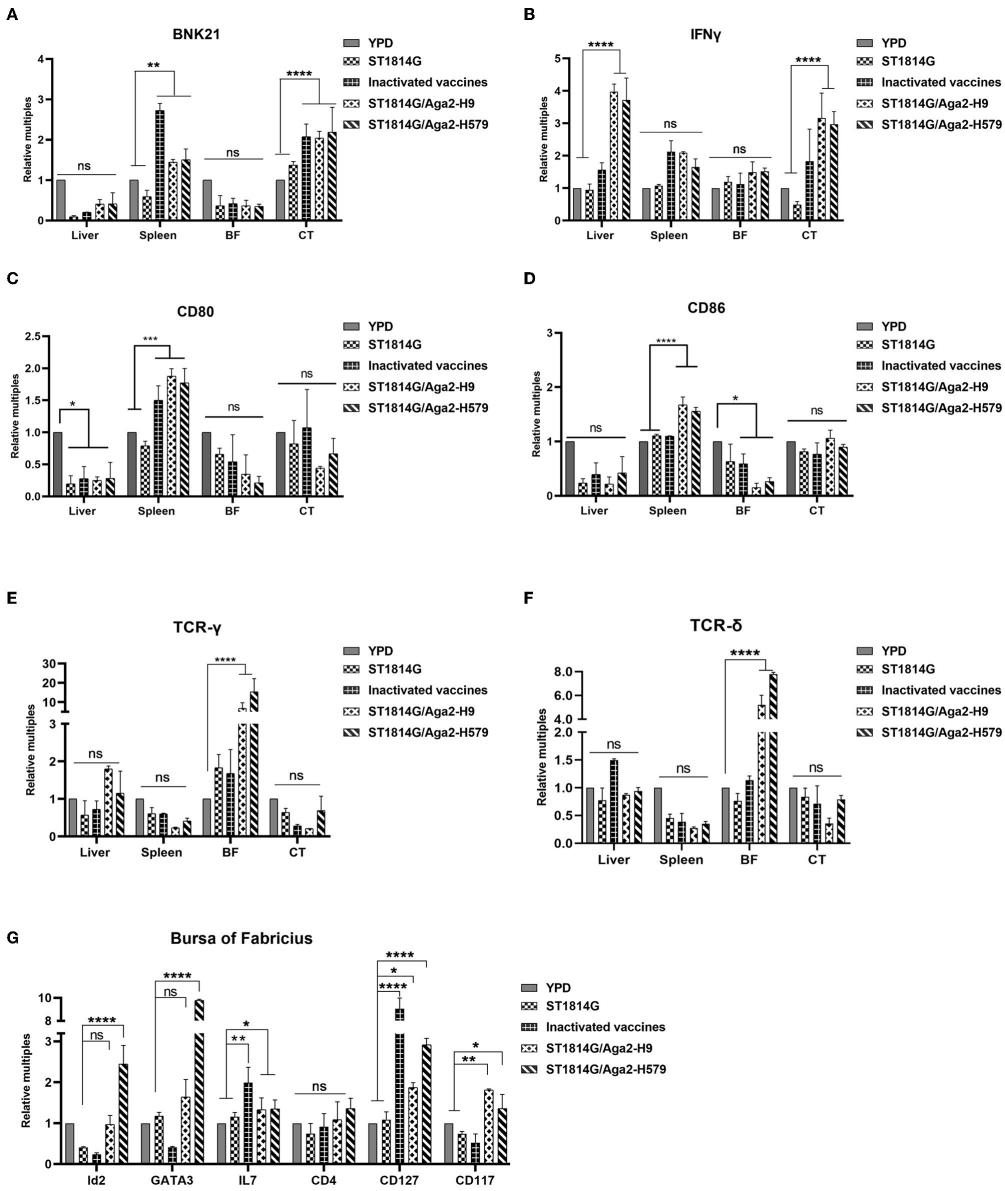
Effect of oral yeast vaccine on the relative mRNA expression of cell factors associated with NK cells, γδT cells, and ILCs. **(A, B)** The mRNA expression of the NK cell surface receptor BNK21 and its secreted IFNG in the five groups of the liver, spleen, bursa of *Fabricius* (BF), and cecum tonsils (CF). **(C, D)** The mRNA expression of the APC cell surface receptor CD80 and CD86 in five groups of the liver, spleen, bursa of *Fabricius* (BF), and cecum tonsils (CF). **(E, F)** The mRNA expression of the gamma and delta chains of T-cell TCR in the five groups of the liver, spleen, bursa of *Fabricius* (BF), and cecum tonsils (CF). **(G)** Transcript levels of ILCs-related factors in the five groups of the bursa of *Fabricius*, including marker molecules, transcription factors, and secreted cytokines. The differences were compared among groups, respectively, and the data in the figures were obtained from three independent experiments and represent the averages ± SD. The significance of differences was determined by a two-way analysis of variance (*p < 0.05; **p < 0.01; ***p < 0.001; ****p < 0.0001 or ns, no significance).

BNK21 is an important molecule marker of natural killer (NK) cells, and IFNγ is the main product of the NK cells, so the expression level of both of them indicated the NK cell abundance to some extent (Yang et al., 2014; Neulen et al., 2015). Our experimental results suggested that both BNK21 (Figure 3A) and IFNγ (Figure 3B) were upregulated in the spleen and cecum tonsils after immunization by inactivated vaccine and oral yeast vaccine, suggesting that NK cells might accumulate in the spleen and cecum tonsils and produce IFNγ in large quantities after immunization with oral yeast vaccine, which are immediately effective in fighting against virus invasion and removing damaged cells.

To monitor the abundance of antigen-presenting cells (APCs), such as macrophage and dendritic cells, we checked macrophage-specific protein CD80 and CD86 and dendritic cell-related chemokine ligand (CX3CL1) and receptor (CX3CR1). Our experimental results suggested that both CD80/CD86 (Figure 3C) and CX3CL1/CX3CR1 (Supplementary Figure 1A) were upregulated in the spleen after immunization by oral yeast vaccine, suggesting that antigen-presenting cells might accumulate in the spleen, which potently activated the NK cells, NKT cells, and T cells, facilitating cytotoxic T-cell responses.

γδ T cells, as tissue-resident T cells, also serve as the first line of defense for immunity and are capable of generating a rapid response to pathogen invasion. To monitor the γδ T-cell abundance, we analyzed the transcription levels of genes encoding the gamma and delta chains of the T-cell receptor (TCR) in different organs of chickens. As shown in Figures 3D, E, the genes were increased in the bursa of *Fabricius*, suggesting that γδ T cells might accumulate in the bursa of *Fabricius* as a primary immune organ.

Innate lymphoid cells (ILCs) are reported to regulate tissue immune homeostasis, resist pathogenic infection, and enhance T- and B-cell-mediated acquired immune responses in humans and mice, however, chicken ILC-like cells have not been fully verified right now. To explore the possibility, we first analyzed and selected the critical and specific homologous genes for human and mouse ILCs and we found that 92.3% of them were cloned successfully (primers for cloning genes showed in Supplementary Table 1), suggesting that chickens, as evolutionarily original animals, are likely to have ILCs. Meanwhile, the transcription factors (GATA3 and Id2), the differentiation-dependent factor IL7, and specific surface markers (CD127 and CD117) of the oral yeast group were all upregulated (Figure 3F, Supplementary Figure 2B) compared to those in the ST1814G group. These are critical factors that regulate the transformation of common ILC precursors (CILPs) to ILCs (Zhong and Zhu, 2017; Zhu, 2017), suggesting that oral yeast-immunized chickens were likely to stimulate more ILC formation by the differentiation of CILPs in the bursa of *Fabricius*.

### Oral yeast vaccine promotes innate immune signaling pathway in the spleen

**Figure 4:**
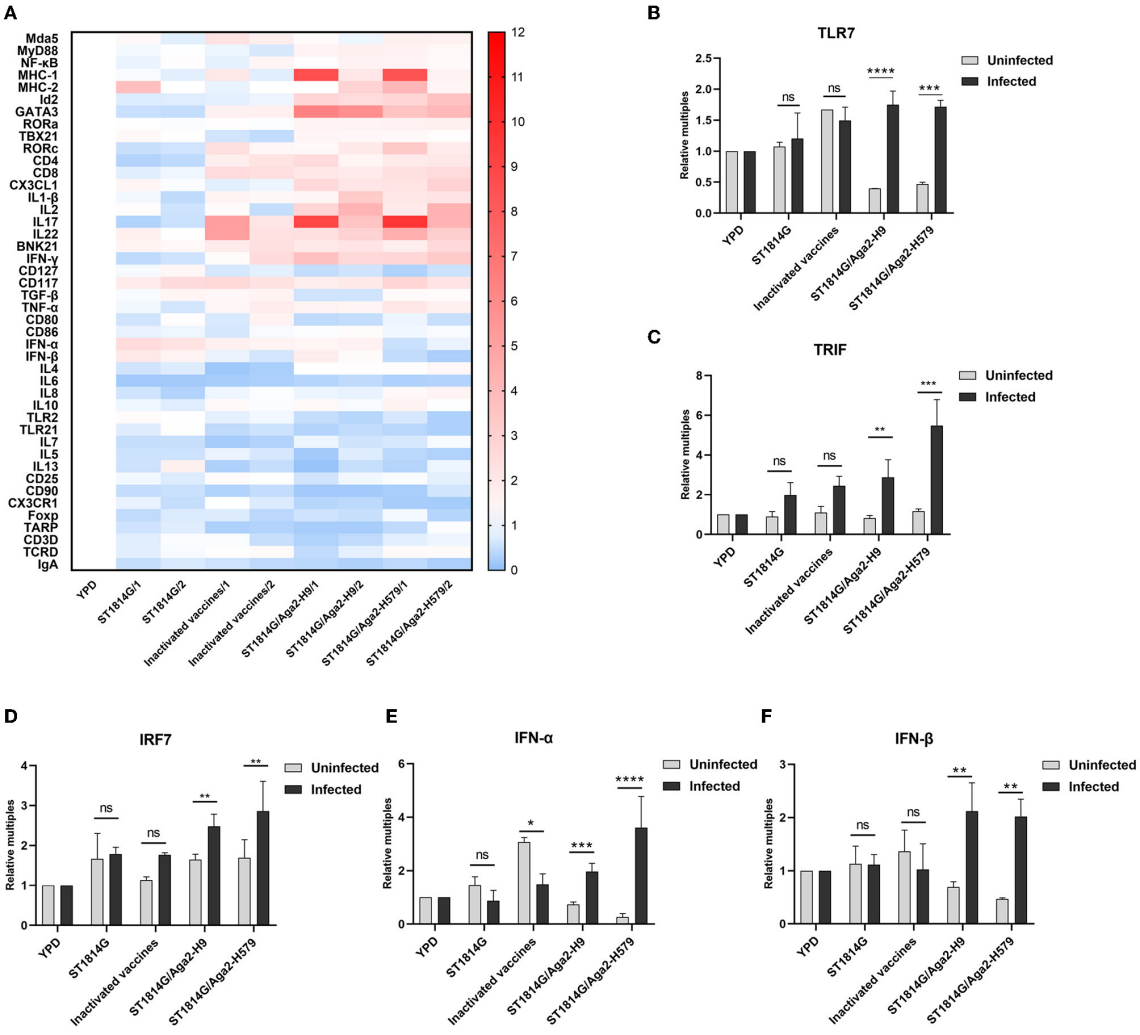
Effect of oral yeast vaccine on the relative mRNA expression of immune-related cytokines in cecum tonsils and signaling molecules of the TLR7 pathway. **(A)** The heat map on transcript levels of five groups of immune-related cytokines in the cecum tonsils (CF). Transcript levels of TLR7 **(B)**, TRIF **(C)**, IRF7 **(D)**, IFNα **(E)**, and IFNβ **(F)** in the spleen of uninfected (gray) and infected (black) chickens in the five groups. The differences were compared among groups, respectively, and the data in the figures were obtained from three independent experiments and represent the averages ± SD. The significance of differences was determined by a two-way analysis of variance (*p < 0.05; **p < 0.01; ***p < 0.001; ****p < 0.0001 or ns, no significance).

In addition to innate immune cells, there are also some important innate immune signaling pathways that constitute the first line of defense against pathogen invasion and mediate the activation of immune cells. To further analyze the protective mechanisms of yeast vaccine-elicited innate immunity, we first examined the transcript levels of factors involved in intracellular innate immunity in different groups (Figure 4A). We found that, compared to the ST1814G group, the factor expression levels were mostly upregulated in cecum tonsils in oral yeast vaccine groups, suggesting that cecum tonsils play an important role in the efficacy of yeast vaccine in chickens. The spleen plays an important role in immune function in the organism, and we found that oral administration of yeast vaccine stimulated TLR7-TRIF-IRF7-dependent interferon signaling pathway in the situation of H9N2 influenza virus infection in the spleen (Figures 4B–F; Supplementary Figure 2A), whereas the expression level of MyD88 was not influenced (Supplementary Figure 1B). Therefore, we concluded that the innate immune signaling pathway might be activated after oral administration of yeast vaccine in the cecum tonsil, which is an important immune organ for fighting against pathogen invasion. Especially, the interferon signaling pathway was triggered a in TLR7-TRIF-IRF7-dependent manner upon virus infection in the oral yeast-immunized chickens.

### Oral yeast vaccine has various effects on different tissue-resident T cell

**Figure 5:**
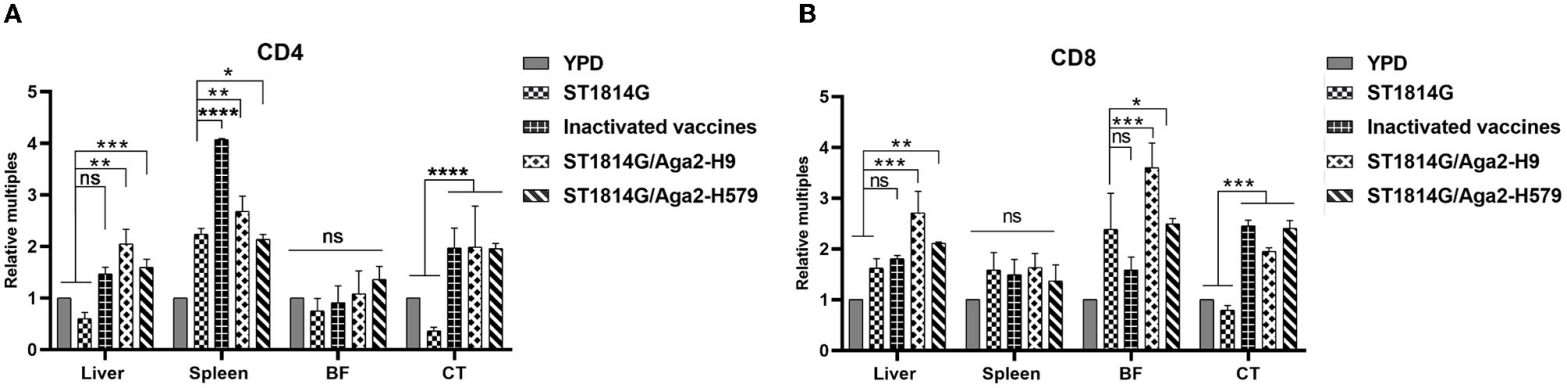
Evaluation of CD4^+^T-cell and CD8^+^T-cell levels after three immunizations. The mRNA expression levels of the five groups of CD4 **(A)** and CD8 **(B)** in the liver, spleen, bursa of Fabricius (BF), and cecum tonsils (CT). The data in the figures were obtained from three independent experiments and represent the averages ± SD. The significance of differences was determined by a two-way analysis of variance (*p < 0.05; **p < 0.01; ***p < 0.001; ****p < 0.0001 or ns, no significance).

As mentioned above, the oral yeast vaccine might cause the accumulation of APCs, such as macrophages and dendritic cells, in the spleen, which implied that the function of T cells might be influenced in the oral yeast vaccination group. Both CD4 and CD8 are characteristic molecule markers of T cells. CD4^+^ T cells mainly perform cellular regulatory functions, promote downstream T helper (Th) cell and regulatory T (TREG) cell differentiation, and achieve a regulatory effective immune response to pathogens. CD8^+^ T cells mainly exert cellular immune functions and secrete perforin and granzyme through MHC-I-dependent processes to protect the organism against intracellular threats such as viruses and bacteria, as well as neoplasms. To explore whether the function of T cells is influenced by the oral yeast vaccine, we next checked the expression levels of CD4 and CD8 in different immune organs and lymph nodes. As shown in Figure 5, the expression levels of both CD4 and CD8 are upregulated in the liver and cecum tonsils. In particular, CD4 is significantly upregulated in the spleen while CD8 is significantly upregulated in the bursa of *Fabricius*. These results indicated that both CD4^+^ T cells and CD8^+^ T cells might play important roles in the liver and cecum tonsils, which was consistent with our above results that the cecum tonsils might be a primary influenced immune organ in the situation of oral yeast vaccination (Figure 4A). In addition, CD4^+^ T cells might dominate immune regulatory responses in the spleen, and CD8^+^ T cells might dominate cytotoxic T-cell responses in the bursa of *Fabricius*.

### Oral yeast vaccine improves the gut microbiota and suppresses the Th17 cell differentiation and IL17-mediated cellular inflammation

It is known that yeast is probiotic to colonize the intestinal microenvironment, and thus, gut microbiota and intestinal mucosal immunity might be changed due to the influence of oral yeast (Zhang et al., 2022b). To further explore the protective mechanism of oral yeast vaccine, we next performed 16S rRNA-amplicon sequencing to analyze the change of gut microbiota in different groups.

**Figure 6:**
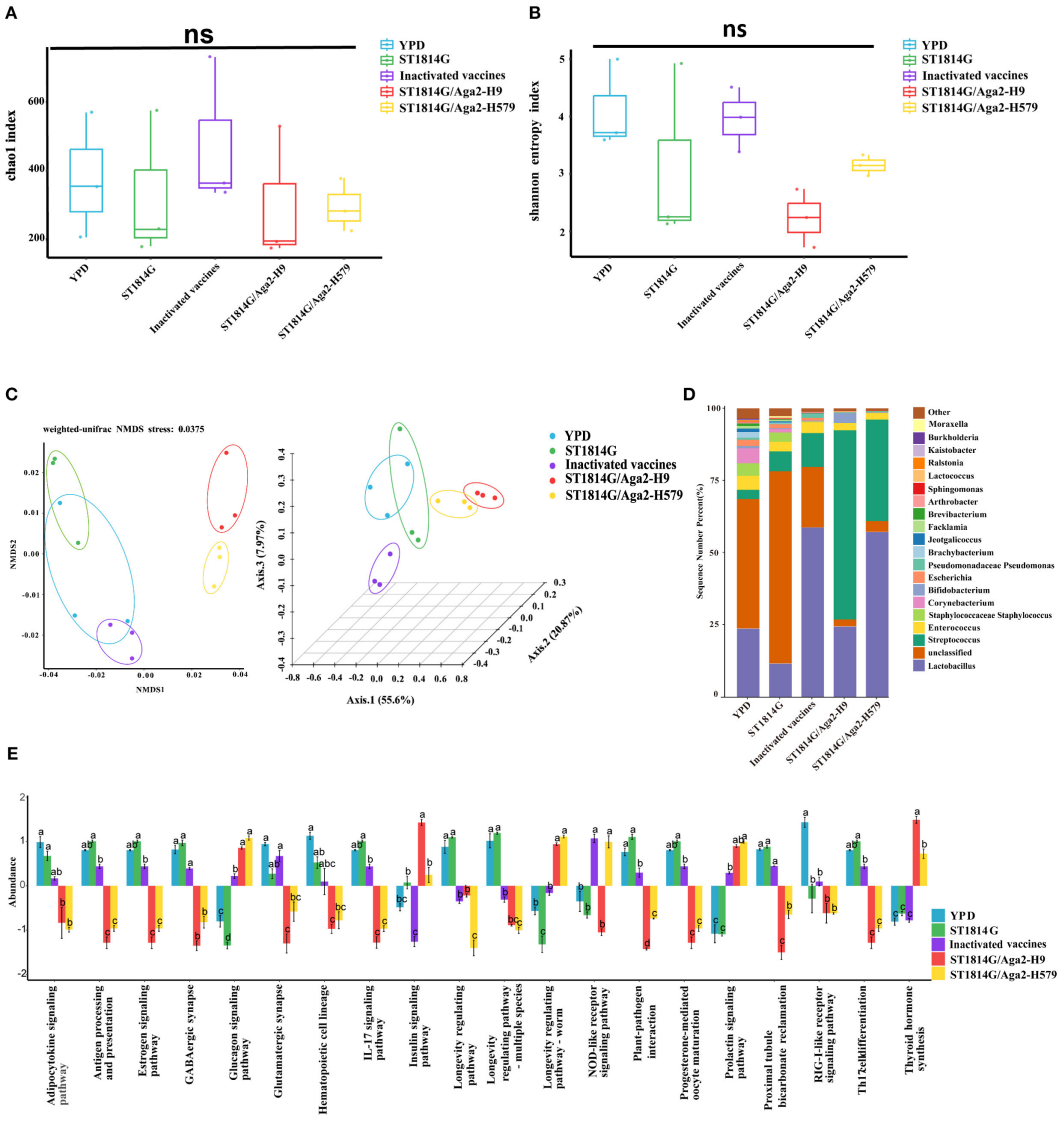
Relative abundance and intergroup diversity of gut microbiota in chickens post-immunization. The alpha diversity index reflects species diversity in the five groups of samples, **(A)** the Chao1 index reflects the species abundance of the 5 groups, **(B)** the Shannon index assesses the species diversity of the 5 groups, **(C)** beta diversity, including NMDS analysis and PCoA analysis, assesses differences in microbial community structure in the five groups of samples. **(D)** Species distribution histogram of chicken gut microbiota in each group at the genus level. **(E)** Analysis of functional differences between groups in the KEGG pathways: Twenty signaling pathways including antigen processing and presentation, estrogen signaling pathway, and GABAergic synapse. The data in the figures were obtained from three independent experiments and represent the averages ± SD. The significance of differences was determined by a two-way analysis of variance or Dunnett's test (*p < 0.05; **p < 0.01; ***p < 0.001; ****p < 0.0001 or ns, no significance).

The alpha diversity index indicates species diversity, namely the abundance and evenness of species composition, of the sample. The Chao1 index and Shannon index analysis reflected that there were no significant differences in species abundance and diversity between the oral yeast vaccine groups and the other groups (Figures 6A, B). The beta diversity index is used to compare species composition between different samples using statistical methods, such as non-metric multidimensional scaling (NMDS) and principal coordinate analysis (PCoA). As shown in Figure 6C, the two oral yeast vaccine groups were much closer to each other, whereas the inactivated vaccine group was closer to the YPD group and ST1814G group, suggesting our oral yeast vaccine indeed had different species composition compared to the yeast vehicle group and the inactivated vaccine group, which might stand for the protective function of oral yeast vaccine in chickens.

Next, we analyzed the relative abundance of species at the genus level of gut microbiota, using the species abundance table from the operational taxonomic unit (OUT) clustering analysis (Figure 6D). Among the eight high abundance genera, compared to the yeast vehicle group and inactivated vaccine group, oral administration of yeast vaccines could increase the abundance of probiotic bacteria (*Bifidobacterium* and *Lactobacillus*), decrease the abundance of pathogenic bacteria (*Escherichia, Corynebacterium, and Staphylococcus*), and maintain normal intestinal bacteria (*Enterococcus* and *Streptococcus*). These changes in gut microbiota indicated that oral administration of yeast vaccines improved the intestinal microenvironment, which might further influence intestinal mucosal immunity through the adjustment of microbial metabolic product.

The different species abundance lists of different groups were further processed for an enrichment analysis based on the KEGG Ontology databases to obtain functional annotations. Notably, among the many different signaling pathways between the oral yeast vaccine group and ST1814G group, the IL17 signaling pathway and T helper 17 (Th17) cell differentiation signaling pathway attracted our attention. The Th17 cells are essential for controlling extracellular bacterial and fungal infections, and they also trigger autoimmune responses by secreting pro-inflammatory cytokine IL17 (Sandner et al., 2021). It is reported that the dynamic balance of the gut microbiome is critical for maintaining Treg/Th17 homeostasis in the intestine, which is important for the susceptibility to inflammatory bowel disease (Omenetti and Pizarro, 2015). The relative abundance of microbiota involved in regulating these two signaling pathways was lower in the oral yeast vaccine group than those in the ST1814G group, suggesting that oral yeast immunization could suppress the Th17 cell differentiation and IL17-mediated cellular inflammation (Figure 6E).

## Discussion

Current strategies for developing and manufacturing the avian influenza vaccine have safety and production issues, are time-consuming, and are somewhat limited in the ability to quickly generate a vaccine matched with mutate virus (Lei et al., 2020). In this study, we successfully developed an oral yeast vaccine candidate that expressed three HA proteins simultaneously for preventing different avian influenza virus subtype infections. Our experimental results suggested that it provided desired protective effect without side effects for chickens. To better understand the protective molecular mechanisms of oral yeast vaccines, we detected the effects of oral yeast vaccine on congenital lymphocyte subsets of different tissues and gut microbiota in different treated groups, such as ST1814G yeast vehicle, traditional inactivated vaccine, and our engineered oral yeast vaccines.

The *S. cerevisiae* surface display system successfully expressed HA proteins, but the measured protein molecular weights were larger than predicted (H5 74.5 kDa, H7 71.5 kDa, and H9 69 kDa). Based on studies reporting that glycosylation in yeast increases the molecular weight of the protein (Liu et al., 2022), we presume that glycosylation occurred during post-translational modification in yeast in this study. It has been proposed that glycosylation can enhance immunogenicity and thermal stability (Maciola et al., 2017) and also can lead to a decrease in protein expression (Peraino et al., 2012). We consider that protein glycosylation in yeast is very complex cellular engineering, and whether it affects various aspects of the biological activity of HA proteins remains to be explored.

The oral yeast vaccine alters the activation dynamics of congenital lymphocyte subsets in different tissues. *S. cerevisiae* is an ideal vehicle for delivering protein subunit and nucleic acid vaccines. β-glucan (Torosantucci et al., 2000) and mannans (Liu Y. N. et al., 2021) from yeast cell walls are the main active components, and the diameter of yeast further facilitates the recognition by antigen-presenting cells of organisms (Silva et al., 2021; Feng et al., 2022). In our study, we found that, compared to the ST1814G yeast vehicle group, oral administration of HA present on the surface of *S. cerevisiae* could upregulate chemokines and the abundance of tissue-resident NK cell, APCs, γδ T cell, and ILCs. In particular, the NK cells might mainly accumulate in the spleen and cecum tonsils while the APCs mainly accumulate in the spleen. In contrast, γδ T cells and ILCs might mainly accumulate in the bursa of *Fabricius* (Supplementary Figure 2B). Those changes in several immune organs and the predominant immune cells in oral yeast birds indicated that the spleen and cecum tonsils could recognize the signaling of HA-displaying yeasty and then continue to trigger signaling pathway of the TLR7-TRIF-IRF7-denpendent interferon signaling pathway after yeast vaccination in the spleen (Figure 4). As the primary immune organ in birds, the activation of innate lymphocytes, such as γδ T cells and ILCs, was observed in the bursa of *Fabricius*, which hints at a possible origin of them from the bursa. The TLR7 can recognize viral single-stranded ribonucleic acid (ssRNA) and promote IL-1β and iNOS production in macrophages of H4N6 LPAIV-infected chicken (Annamalai et al., 2015; Abdul-Cader et al., 2018). The boosted TLR7-TRIF-IRF7-dependent interferon pathway and IL-1β production after the virus challenge maybe contribute to the deducing viral loading and good protection observed in our oral yeast vaccine. The existence of a memory response by oral immunity in birds and how they work is still a challenging problem.

The oral yeast vaccine prompts ILC3 cell differentiation in the bursa of *Fabricius*. The enhanced CILPs differentiated into ILCs were deduced in the bursa of *Fabricius in* oral yeast-immunized chickens and were supported by the upregulation of the specific transcription factors (GATA3, ID2), essential regulatory cytokine (IL7), and surface molecule markers (CD127, CD117) (Figure 3G). To understand which type of ILC cells contributes to it, the mRNA levels of three subtype-specific molecular markers of ILCs in the bursa tissues were detected. Both ILC1- and ILC2-related transcription factors and cytokines (T-bet and IFNγ for ILC1; RORα, IL5, and IL13 for ILC2) (Vivier et al., 2018; De Pasquale et al., 2021) were suppressed or detected no difference among the groups. In contrast, ILC3-related transcription factors RORγt were significantly upregulated (Supplementary Figure 1C). Meanwhile, IL22 secreted by ILC3 was also significantly increased in the bursa of *Fabricius*. These results suggested that the oral yeast vaccine elicits ILC3 proliferation to promote the innate immune response, mucosal repair, and barrier establishment against extracellular pathogen infection. Certainly, there is an urgent need for the successful sorting and identification of ILC cells in chickens to further verify their functions.

Oral HA-present *S. cerevisiae* is a potent alternative vaccine causing ideal protective responses and mild inflammatory responses. Usually, the inactivated vaccine triggers a strong inflammatory response or macrophage infiltration that leads to a relatively big liver index. However, this phenomenon was not induced in the groups of oral yeast vaccine (Figure 2B). To understand the contribution of the engineered yeast vaccines in the inflammatory response, the major pro-inflammatory factors, including IL6, IL8, TNF-α, and TGF-β, and the suppressing inflammatory cytokine IL10 (Liu Z. et al., 2019) were measured. The downregulation of all pro-inflammatory factors was observed in the organs of oral yeast groups compared with those in the inactivated vaccine group, which was consistent with the above organ index experiments (Supplementary Figures 3A–D). Interestingly, IL10 was significantly upregulated in the spleen after immunization. Considering the accumulation of APCs in the spleen, we speculated that specific APCs, such as macrophages, might be dynamically regulated by IL10 to perform the inhibition role of the inflammation response *in vivo*. Yeast is able to colonize the mouse intestine and become the predominant species further reshaping the microbiota and intestinal microenvironment (Dong et al., 2022). The functional prediction of gut microbiota demonstrated a reduced Th17 cell/IL17 signaling pathway in the yeast vaccine group, which also decreased the intestinal inflammation response (Zhang et al., 2022a). Taken together, these results indicated that oral administration of yeast vaccine might facilitate the repairmen of gut mucosa damage caused by pathogen invasion, which elicit the innate intestinal mucosal immunity to protect the organism from fatal influenza virus infection (Ravindran and Thornton, 2022; Serafini et al., 2022).

**Figure 7:**
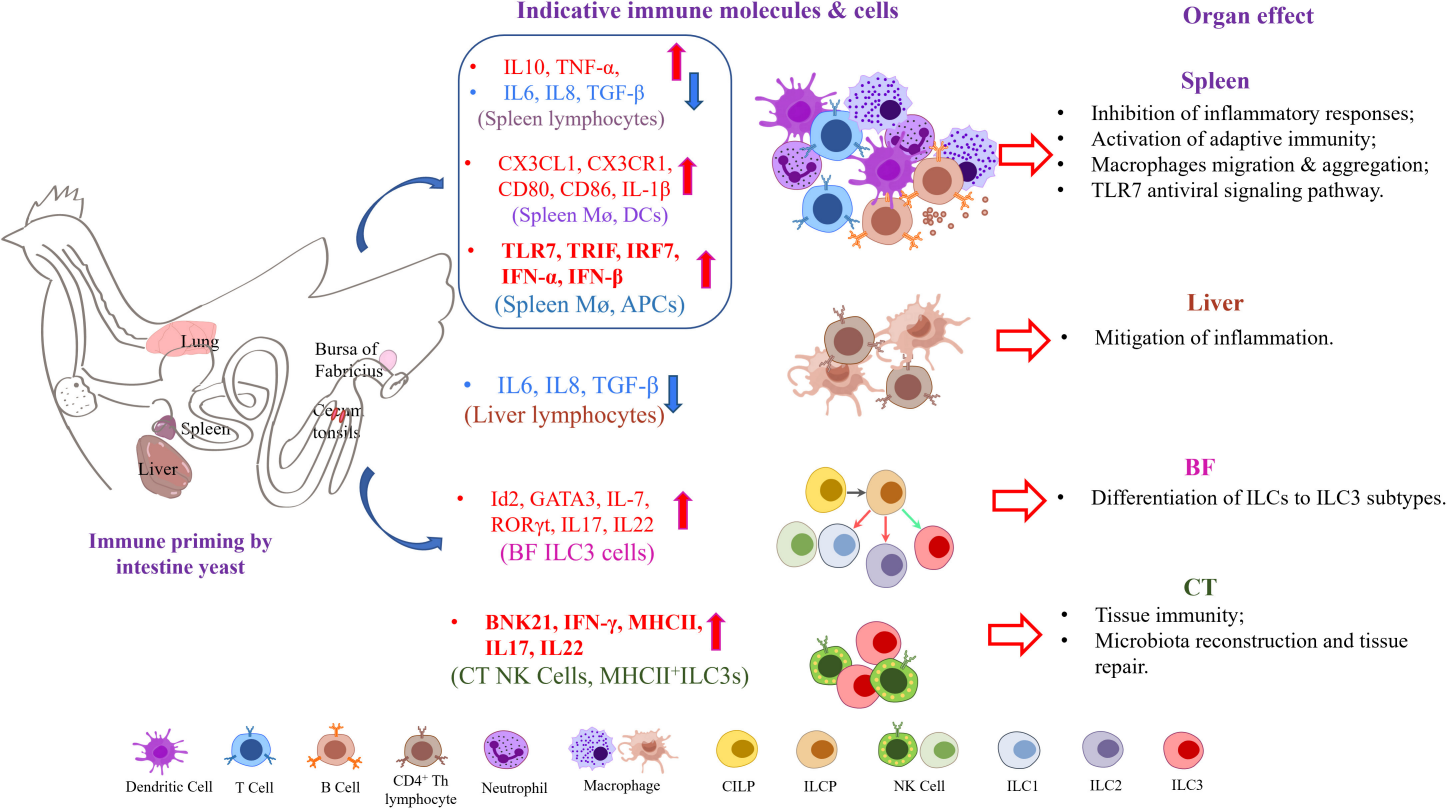
Oral yeast vaccine caused alterations of primary immune cytokines in several immune organs and lymph nodes in chickens.

Generally, the roles of oral yeast vaccine in defending avian influenza virus infection may be performed by altering immune response in different immune organs or lymph node tissues (Figure 7). 1. The activation of the APC-indicated chemokines and TLR7-TRIF-IRF7-dependent interferon signaling in the spleen indicates the crucial roles of the spleen in response process of oral yeast immunity. 2. The NK cells, γδ T cells, and ILC3 cells derived from CILPs from the bursa change the cytokine spectrum and trigger the activation of some immune cells, such as CD4+ T cells in the spleen and CD8+ T cells in the bursa of *Fabricius*. 3. The oral administration of yeast vaccine alters the structure of gut microbiota and their metabolites and stimulates a mild inflammatory response, which is important to the contribution of their good immune protection.

Further studies will be required to fully elucidate the mechanisms by which the HA yeast vaccine provides protection. In most cases, symptoms and severity are greatly reduced, and the virus is cleared faster in yeast-feeding birds. Our present findings suggest that protection against IAV infection may be provided through non-neutralizing mechanisms, such as innate immune cell function regulation and immune microenvironment homeostasis remodeling. The deficiency in our study is that, in order to construct a multivalent yeast vaccine, we constructed an *S. cerevisiae* strain of H579, but limited by the existing laboratory conditions, we only did the attack protection experiment for H9. We believe that H5 and H7 are also expressed, so they can be resistant to all three viruses to some extent, but this needs further testing. Our overall approach will likely be useful for infectious diseases other than influenza viruses. Multivalent yeast vaccines may be applied against other variable pathogens. Additional studies will be required to reveal the function of those novel innate lymphocytes, such as macrophages, NK cells, γδ T cells, and ILC3 cells, which participated in immune protection and understand their underlying immunological mechanisms in detail.

## Materials and methods

### Virus, vaccine, yeast, and plasmids

The H9N2 subtype avian influenza virus strain (GenBank: KC296446.1) was isolated and stored in our laboratory. The avian influenza-inactivated vaccine was bought from YEBIO Bioengineering Co., Ltd., Qingdao (Qingdao, China). In this study, yeast strain ST1814G constructed in our laboratory (MATa aga1 his3Δ200 leu2Δ0 lys2Δ0 trp1Δ63 ura3Δ0 met15Δ0) was utilized as the host cell for the surface display system. Plasmids for constructing recombinant yeast strains include nutrient-deficient screening markers: PMV- LEU2, PMV- TRP, PMV- HIS, and homologs chromosome armsPMV-URR1, PMV-URR2 and performed the element assembly as previously described (Guo et al., 2015). The POT-GPD-RFP-ADH1 plasmid containing 667 bp GAPDH promoter sequences, 187 bp ADH1 terminator sequences, and RFP selective marker sequence was from our laboratory.

### Construction of transcription units

The chimeric H5/7/9 HA (hemagglutinin) recombinant yeast vaccine was constructed, the 1404bp sequences of the H5N8 HPAI virus strain (A/goose/Hebei/HG12/2021), the 1515bp sequences of the H7N9 HPAI virus strain (A/duck/Jiangxi/3096/2009), and the 1344bp sequences of the H9N2 LPAI virus strain (A/Hebei/218/2010) were selected. HA-optimized genes (with the transmembrane domain and the signal peptide removed) (H5N8 subtype and H7N9 subtype) were cloned from laboratory strains, and the HA gene sequence of the H9N2 subtype of AIV was obtained from the NCBI website and optimized for synthesis by GENEWIZ (Beijing, China). Three HA genes were subcloned into three yeast expression vectors (pGPD-ADH1-POT with His-tag at the C terminal and Aga2 signal peptide at the N terminal), respectively by using a One-step Cloning kit (Vazyme, Nanjing). These strategies would anchor the recombinant proteins to the yeast surface and allow tracking of expression by anti-His-tag specific antibodies. The recombinant plasmids were named pGPD-H5-ADH1-POT, pGPD-H7-ADH1-POT, and pGPD-H9-ADH1-POT, respectively.

### Construction of yeast

Using yeast that had completed the construction of the H5-Leu module on chromosome IV as a substrate, the H7 gene was constructed into chromosome XVI using the filter tag Trp and the H9 gene was constructed into chromosome III using the filter tag His (Figure 1A). Our transcriptional construction unit refers to a previously published article, briefly described as follows: Construct pGPD-HA-ADH1-POT vector, *Bsa*I enzyme cleavage plasmid to obtain linearized transcript; perform *Bsm*BI enzyme cleavage with screening marker plasmid, URR1, URR2; then T4 ligation, transformation into yeast in LiAc, screen positive clones corresponding to nutrient-deficient plates, and extract genome for identification. H5/H7/H9 are all using the same method.

### Western blotting

The expression of HA protein in trivalent recombinant yeast was identified by the Western blotting technique. In total, 2 ml of yeast cultured overnight at 30°C was taken at 10,000 rpm for 1 min, which was then collected. The pellets were broken with an equal amount of silica beads of 60 μL protein extraction buffer (2 % SDS, 100 mmol/L DTT, 125 mmol/L Tris-HCl, pH 6.8). Next, the mixture was centrifuged at 12,000 rpm for 10 min at 4°C, and the supernatant was carefully obtained into a new EP tube by adding 5× loading buffer and boiled for 10 min. Treated samples were resolved on a precast 12% polyacrylamide gel and then electrophoretically transferred to the methanol-activated polyvinylidene fluoride membrane (PVDF) (PALL, USA), after blocking with TBS-Tween containing 5% skim milk at room temperature for 2 h and then incubated with a rabbit anti-His antibody (1:5,000 diluted, Yeasen Biotech, Shanghai, China) at room temperature for 1 h. Following three 5-min washes with TBS-Tween, the membrane was followed by 1:5000 diluted horseradish peroxidase (HRP)-conjugated goat anti-rabbit IgG, (Yeasen Biotech, Shanghai, China) for 1 h and then treated with three final washes. Specific proteins were visualized with Western lightning chemiluminescence reagent plus substrate mixture (Bio-Rad, USA) and imaged using the ChemiDoc imaging system (Gel DocTM XR + imaging system, Bio-Rad, USA).

### Immunofluorescence and flow cytometry

The yeast cells containing the target genes were harvested and centrifuged. The blank strain of ST1814G was used as a negative control during this experiment. First, the yeast cells were centrifuged at 6000 rpm for 1 min at 4°C. The cells were treated with a mouse anti-His-tag and followed by FITC-conjugated rabbit anti-mouse IgG antibody at 4°C for 1 h and re-suspended with 500 μL of sterile PBS. Finally, 5 μL of *S. cerevisiae* pellets were used for immunofluorescence assay under an inverted fluorescence microscope. For flow cytometry analysis, 500 μL pellets were analyzed by flow cytometry analysis (BD FACSCalibur, BD Bioscience, San Jose, CA, USA).

### Oral immunization and virus challenge

The animal study was performed following the guidelines and regulations of the Laboratory Animal Ethical and Welfare Committee of Tianjin University (permit number, TJUE-2021–051). A total of 65 (mixed sex) egg-laying chickens (White Laiheng) were obtained and housed in specific pathogen-free (SPF) facilities. All birds were given free feed and free access to drinking water. The chickens were divided into five groups (13 birds per group) named YPD group, ST1814G group, inactivated vaccine group, ST1814G/Aga2-H9 group, and ST1814G/Aga2-H579 group. Immunization methods are presented in Table 1.

**Table 1:** Experiment design of chicken immunization.

**Groups**	**Number of chickens**	**Dosage per chicken**	**Immunization**	
			**Times**	**Route**
YPD	13	1 mL	3	Oral administration
ST1814G	13	1 × 10^9^ CFU	3	Oral administration
Inactivated vaccines	13	0.2 mL	1	i.m.
ST1814/Aga2-H9	13	1 × 10^9^ CFU	3	Oral administration
ST1814/Aga2-H579	13	1 × 10^9^ CFU	3	Oral administration

At 5 days after each immunization, the weight of three randomly selected poultry was taken, and blood and anal swab samples were taken for ELISA assays to detect changes in antibody titers in each group. On day 5 after the third immunization, three chicks were randomly euthanized for tissue sampling (liver, spleen, lung, bursa of *Fabricius*, and cecum tonsils) to detect the changes occurring in the different tissues. On day 6 after the third immunization, seven birds per group (mixed sex) were randomly selected and challenged intranasally with 100 μL A/Hebei/218/2010 (H9N2) virus (TCID_50_ = 10^−4.8^/0.1 mL) and observed for 12 days to record the mortality rate, while blood was collected on the 4th, 7th, and 10th days to determine the virus titer in blood. The animal immunization and the virus challenge process are shown in Figure 1B. At the end of the experimental trials, the birds were humanly euthanized and adequately disposed of.

### Analysis of gut microbiota

Sampling was performed on the 5th day after the end of immunization, and the intestinal contents were taken from 10 cm above the junction of the small intestine and the cecum of three chickens in each group. Samples were sent to Wekemo Tech Group Co., Ltd. (Shenzhen, China) for 16 S rRNA sequencing. The pair of primers (Forward: 5′ -ACTCCTACGGGAGGCAGCA- 3′ and Reverse: 5′ -GGACTACHVGGGTWTCTA AT- 3′) were obtained according to the V3–V4 conserved region. The amplicon analysis process is based on Qiime2 software, and amplicon second-generation sequencing data are de-noised to generate ASV, taxonomic annotation, species screening, basic statistics, significant difference comparison, alpha diversity analysis, beta diversity analysis, correlation analysis, PICRUSt2 functional prediction, functional statistics, functional difference comparison, etc. Among Dunnett's multiple comparison tests, the KEGG Orthology (KO) abundance table was selected for functional difference comparison, and the Organismal Systems was selected among the seven classes of biometabolic pathways. All data were processed using the online platform of Wekemo Bioincloud (https://www.bioincloud.tech).

### Enzyme-linked immunosorbent assay (ELISA)

HA-specific serum IgG and mucosal IgA antibody levels were separately determined by ELISA. In brief, 96-well ELISA plates were coated overnight at 4°C using 0.1 μg of recombinant HA protein of A/Hebei/218/2010 (H9N2) as the antigen. The wells were washed three times with PBS containing 0.05% Tween 20 (PBS-T) and blocked with PBS-T containing 1% BSA for 2 h at room temperature. To the reaction add serum at 1:40 dilution (select optimal dilution after serial dilutions) or the anal swab in PBS to the plate and incubate at 37°C for 1 h, followed by incubation with HRP-goat anti-chicken IgG conjugate (Solarbio, Beijing, China) or goat anti-chicken IgA conjugate (Abcam, USA) at 37°C for 1 h, respectively. After incubation with TMB substrate solution (Solarbio, Beijing, China) for 12 min in the dark, the reaction was blocked by 2 M H_2_SO_4_, and the optical density (OD405) of each well was measured using a microplate spectrometer.

### Quantitative real-time PCR (qRT-PCR)

The expressions of chicken immune-related cytokine genes in the inactivated vaccine group, the oral yeast vaccine group (ST1814G/Aga2-H9 and ST1814G/Aga2-H579), the control ST1814G group, and the blank control YPD group were detected by quantitative PCR (qPCR). For each group of three, RNA was extracted from the tissues using the TRIzol reagent (Yeasen Biotech, Shanghai, China), reverse transcription was performed using the Hifair^®^III 1st Strand cDNA Synthesis SuperMix (Yeasen Biotech, Shanghai, China), and the concentration of cDNA generated was ensured at 2,000 ng/ul. For qPCR, primers for CD4, CD8, CD127, CD117, GATA3, etc., were designed using the Primer-Blast tool (Table 2). The absolute fluorescence quantitative PCR assay was used to quantify the viral titer of H9N2, detecting primers in Table 2. qPCR was performed in a total volume of 20 μL per well, using the Hieff^®^ qPCR SYBR Green Master Mix (Yeasen Biotech, Shanghai, China) and the amplification step consisted of 40 cycles of pre-denaturation at 94°C for 5 min, followed by denaturation at 94°C for 10 s and extension at 60°C for 30 s, followed by melting curve analysis. CT values were determined in triplicate for each sample using an ABI 7500 real-time quantitative fluorescence PCR instrument. Data were calculated based on the 2-ΔΔCT method. β-actin was used as an internal reference for the normalization of relative mRNA expression, and the error lines indicated the standard.

**Table 2:** Primers for qRT-PCR.

**Gene name (chicken)**	**Primer sequence(5′-3′)**	**Size (bp)**	**References**
H9-HA	F:CAGAACAAGAAGGCAGCAA R:AATGTGATGACCATTGCATGG	199	GenBank: KC296446.1
CD4	F:AGGATTGTGGAACTGTCACCTC R:CTGCCACCTCATACCAGTGATT	174	GenBank: DQ202315.1
CD8	F:AGCCACAACAACAGCAGCAC R:TACAAGGAGCACGAGGCAGA	177	GenBank: AY519197.1
CD80	F:CAGCAAGCCGAACATAGAAAGA R:AGCAAACTGGTGGACCTGAGA	270	NM_001079739.2
CD86	F:AAGGATACCAGATACCCTCCCT R:AGGGTGATTGCCAGAAAGCC	189	XM_046908563.1
CX3CL1	F:TCTCCAGATCCCGTTTGCAC R:CACGATCTTCTCCACCCACG	241	GenBank: AM398231.1
CX3CR1	F:ACCACAGGGAGTCGCATTTA R:TCCAGCAAGTGCGGTTACTTT	198	XM_040696048.2
IL1-β	F:CCAGAAAGTGAGGCTCAACATTG R:GACATGTAGAGCTTGTAGCCCTT	114	GenBank: HQ329098.1
IL10	F:TTCTTCCCGTAACCACGTCC R:AGGCAGTCATGCGTTGTTTG	193	NM_001004414.4
IL6	F:CCTGTTCGCCTTTCAGACCT R:GGGATGACCACTTCATCGGG	171	GenBank: HM179640.1
IL8	F:ATTCAAGATGTGAAGCTGAC R:AGGATCTGCAATTAACATGAGG	196	GenBank: DQ393272.2
TNF-A	F:CCGCCCAGTTCAGATGAGTT R:GCAACAACCAGCTATGCACC	130	GenBank: AY765397.1
TGF-B	F:GGTGCCCCATCGGAGTTATT R:TTGCTGAGGATTTGACCCCG	186	XM_040694846.2
BNK21	F:GGGAAGGCAAAGAGCATCCA R:CTCCAGAAATGCTCCCGAGG	138	XM_046928526.1
TARP	F:TGCCTACTGGGAGTCACGAT R:TCATCGGTCCATTTCACCCG	195	NM_001318455.1
TCRD	F:GGTACCGTCAGACACTCGAT R:TACAGGGCACGTAGAGACAGA	154	GenBank: AF175433.1
Id2	F:CTGACCACGCTCAACACAGA R:GTTAGCGTGGATTCCTCCCC	272	NM_205002.2
GATA3	F:GTTCGGGTGCAAATCACGAC R:GATCGCCGTTGGCATTTCTC	272	XM_046908458.1
IL7	F:GGACCATGTCCCATGCCTT R:GCTCTTCGATGTCATGGCTG	156	GenBank: AJ852017.1
CD127	F:AGGATCAAGCCTGTCGTGTG R:TCGGCTTTTGCTTGAATGCC	158	NM_001080106.1
CD117	F:GTTGAAACCAAGCGCCCATT R:AATGAATCTCGCTTCCGCCT	195	NM_204361.1
TBX21	F:GACCTCTACGACAAGGAGCC R:CTCAGCTCCAGCCCCAGG	153	XM_040692014.2
RORα	F: AGCTCGCATCCCATCTGGA R:CGGAGCGGACTCCATGTTTT	144	XM_046899012.1
RORγt	F:CCACAGACCGCCATGCGAG R:GGACATGCGGCCAAACTTGA	270	XM_025143434.2
IL5	F:CAGTGCTGGCTCACATTCAG R:CTGTATCCACCCTTCTGCGG	203	NM_001007084.2
IL13	F:AGTCCCTGAGCATTTGGGTG R:CAACTGTGGGGATGAAGGCA	230	NM_001007085.3
IL17	F:GATGCTGGATGCCTAACCCA R:TGTTTGATGGGCACGGAGTT	257	GenBank: AY744450.1
IL22	F:AGCCCTACATCAGGAATCGC R:CAGGGATGCCAGGAACTGTG	230	NM_001199614.1
IFNα	F: CACCTTCCTCCAAGACAACGATT R:GGCATCCAGCATCTCCTTTC	130	GenBank: GU119896.1
IFNβ	F: CCTCAACCAGATCCAGCATT R:GGATGAGGCTGTGAGAGGAG	180	GenBank: KF741874.1
IFNγ	F: CTGACAAGTCAAAGCCGCAC R:GCATCTCCTCTGAGACTGGC	230	GenBank: AH009942.2
TLR7	F:TGTGATGTGGAAGCCTTTGA R:ATTATCTTTGGGCCCCAGTC	219	NM_001011688.3
TRIF	F:GGTGCCACATTCTGTGAGGA R:GAAGGTCGCTGGATGGACTC	173	NM_001081506.1
MyD88	F:GAAAGAAGGTGTCGGAGGATG R:AATTGTAATGAACCGCAAGATACT	183	NM_001030962.5
IRF-7	F:AAGTGCAAGGTCTTCTGGGC R:GGAAGATGGTGAAGTCGGGG	172	NM_205372.2
β-actin	F:ATGAAGCCCAGAGCAAAAGA R:GGGGTGTTGAAGGTCTCAAA	120	GenBank: L08165.1

### Statistical analysis

A pairwise comparison between treatment and control groups was performed using statistical tools analysis of variance (ANOVA) using SPSS software, version 18.0 (SPSS, Chicago, IL, USA). All the graphs were generated using GraphPad Prism 5.0. (GraphPad Software, La Jolla, CA, USA) “ns” indicates no significant difference (*P* > 0.05). “^*^,” “^**^,” and “^***^” indicate statistically significant differences with values of *P* < 0.05, *P* < 0.01, and *P* < 0.001, respectively.

## Data availability statement

The datasets presented in this study can be found in online repositories. The names of the repository/repositories and accession number(s) can be found in the article/Supplementary material.

## Ethics statement

The animal study was reviewed and approved by the Laboratory Animal Ethical and Welfare Committee of Tianjin University. Written informed consent was obtained from the owners for the participation of their animals in this study.

## Author contributions

JH conceived and designed the experiments, HanZ, ZL, HuiZ, YG, XZ, LZ, LY, SL, CL, DC, RX, and YL performed the experiments, HanZ, ZL, and JH analyzed the data and wrote the study. All authors contributed to the article and approved the submitted version.
