# Supplementary material for: Recombinant hemagglutinin displaying on yeast reshapes congenital lymphocyte subsets to prompt optimized systemic immune protection against avian influenza infection
Source: Front Microbiol. 2023 May 31;14:1153922. doi: 10.3389/fmicb.2023.1153922 (PMC10264594; doi:10.3389/fmicb.2023.1153922)
Supplement: Supplementary file 1 [file Data_Sheet_1.docx]

**Supplementary materials**


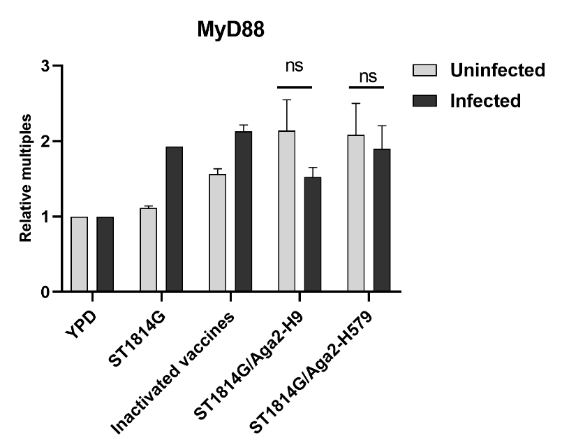

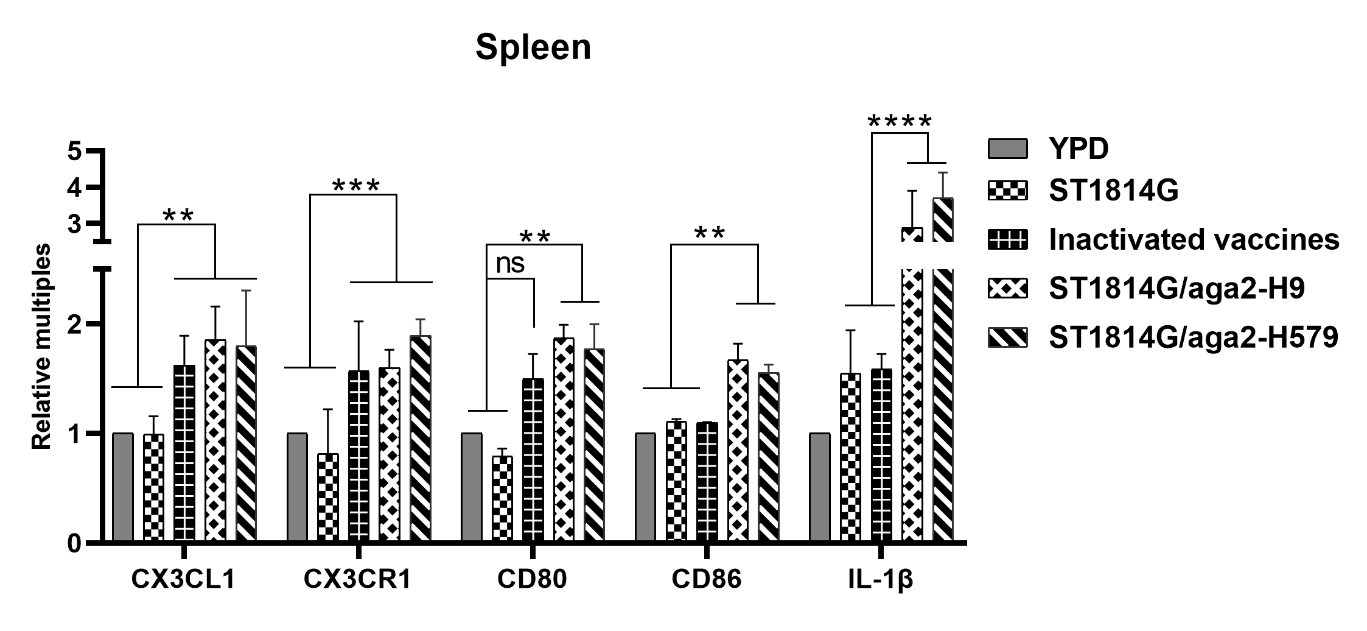


**A**

**B**


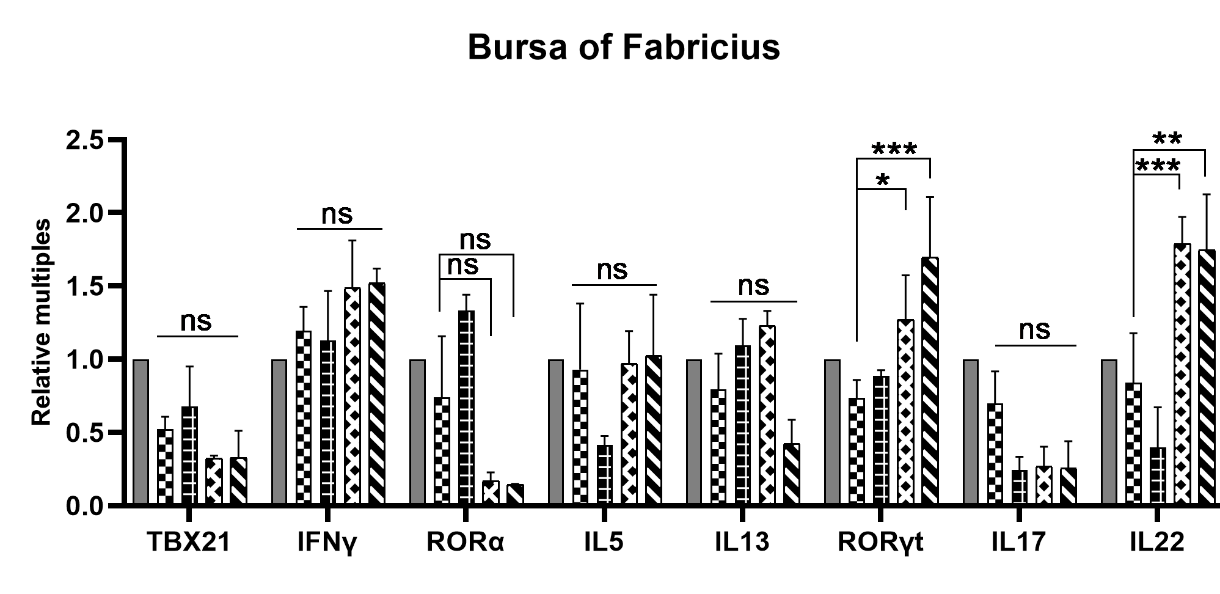


**C**


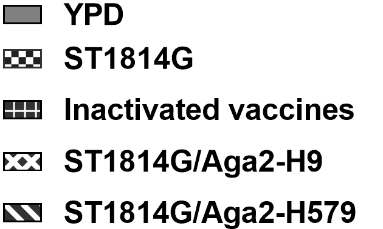


**Supplementary Figure 1. The transcript levels of five groups of immune-related cytokines.** (A) The mRNA expression of the Macrophage chemotactic factors CX3CL1, and its receptor CX3CR1, and IL1-β in five groups of Spleen. (B) Transcript levels of MyD88 in the spleen of uninfected (gray) and infected (black) chickens in five groups. (C) Transcript levels of essential transcription factors and cytokines in three subtypes of ILCs (TBX21, IFNγ for ILC1; RORα for ILC2; RORγt, IL17 and IL22 for ILC3).


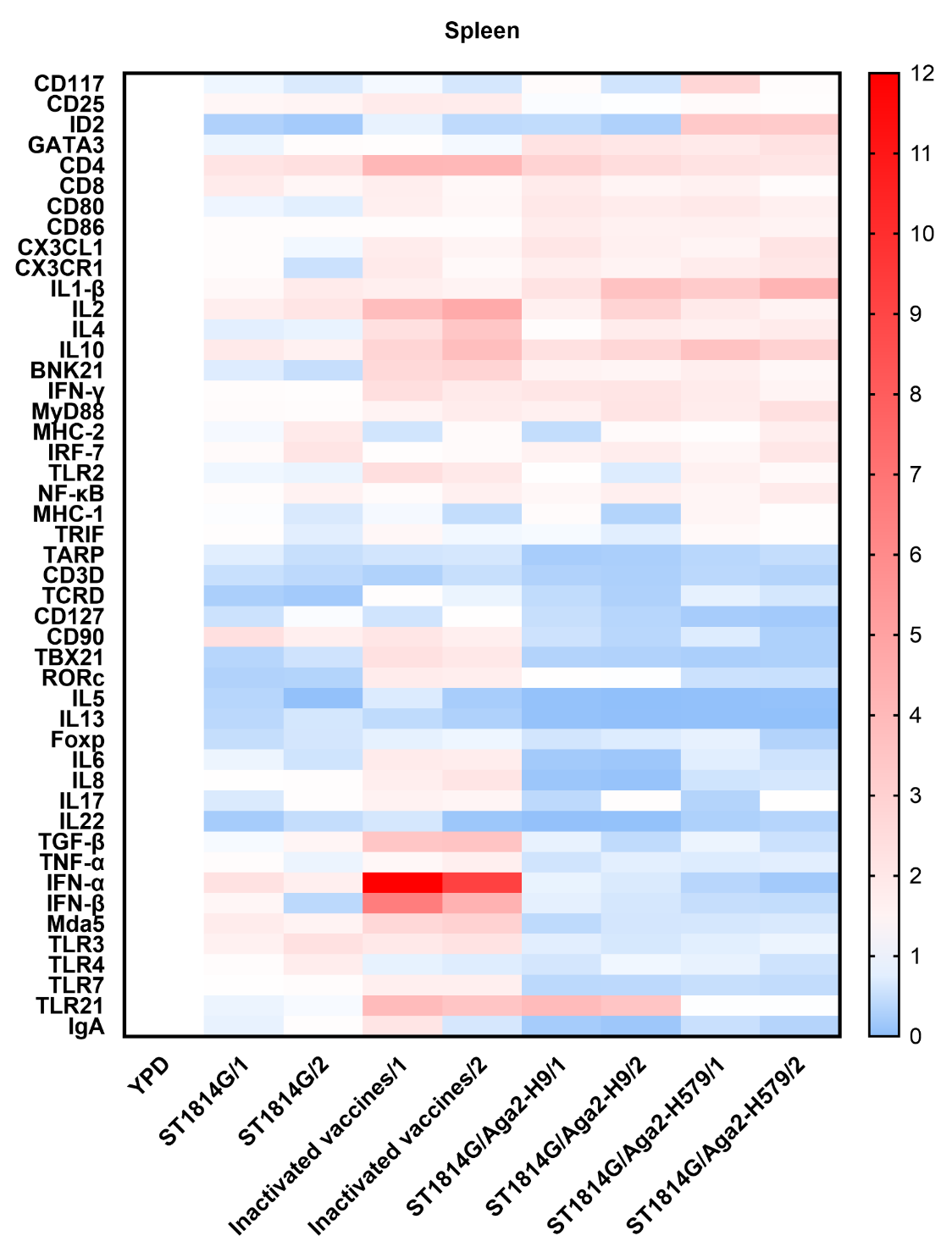

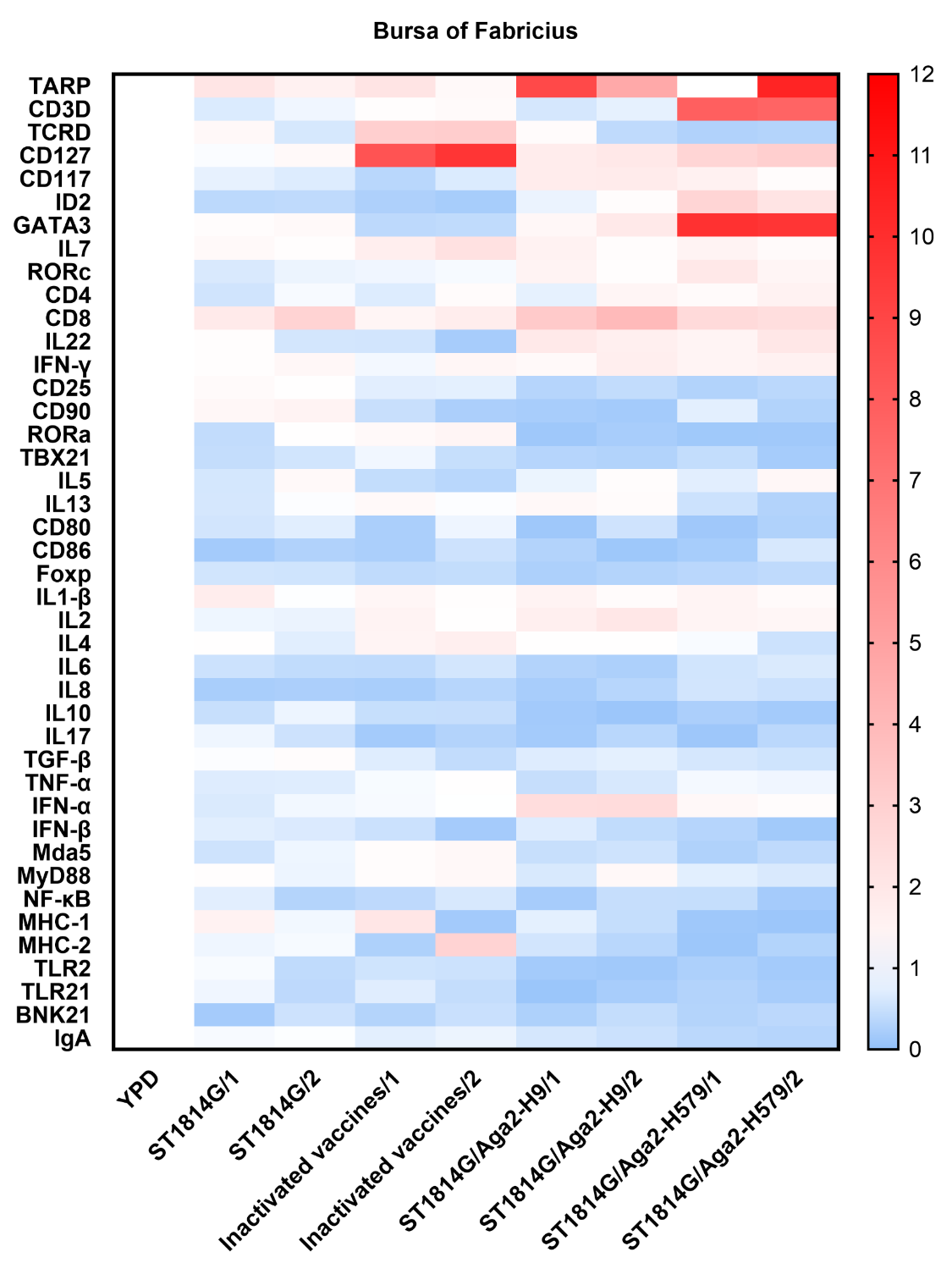


**A**

**B**

**Supplementary Figure 2. Effect of oral yeast vaccine on the relative mRNA expression of multiple immune-related cytokines in Spleen and Bursa of *Fabricius*.** (A) Heat map on transcript levels of five groups of immune-related cytokines in Spleen. (B) Heat map on transcript levels of five groups of immune-related cytokines in Bursa of *Fabricius*.


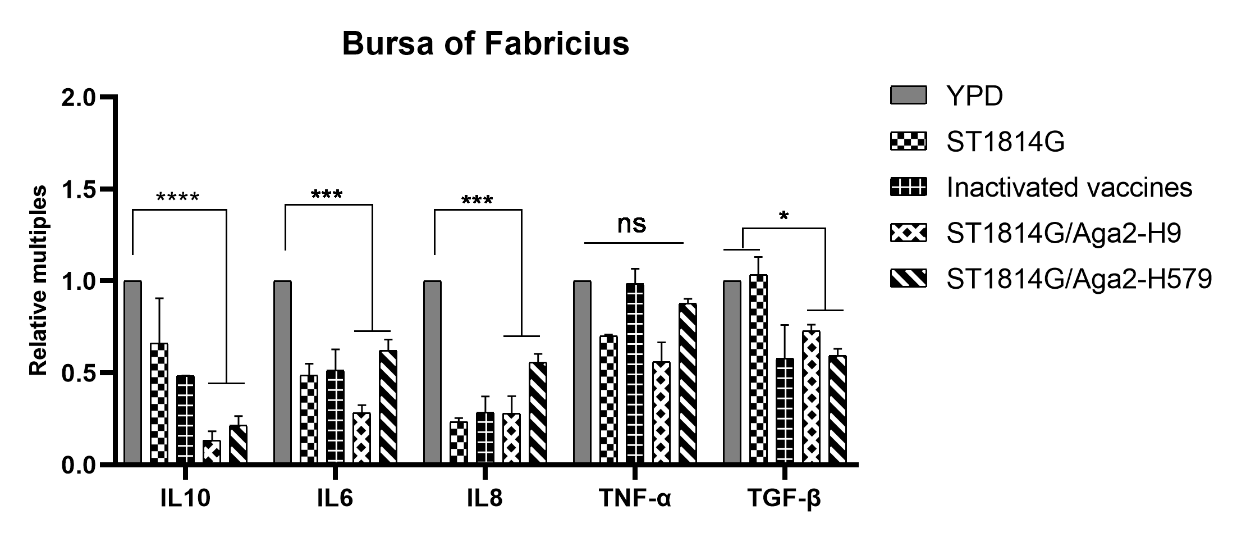

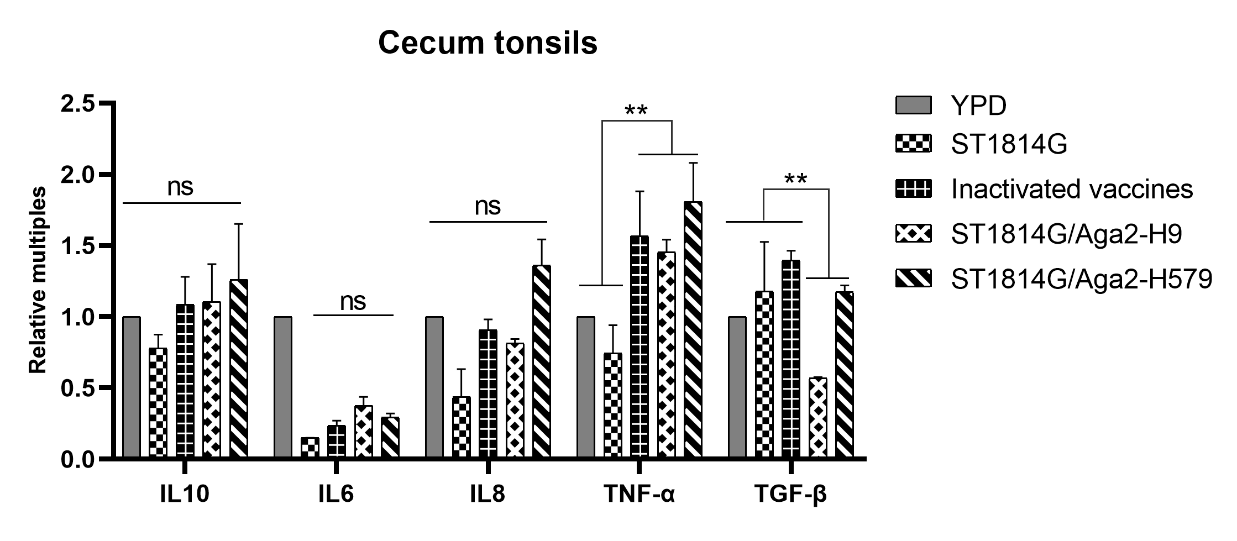

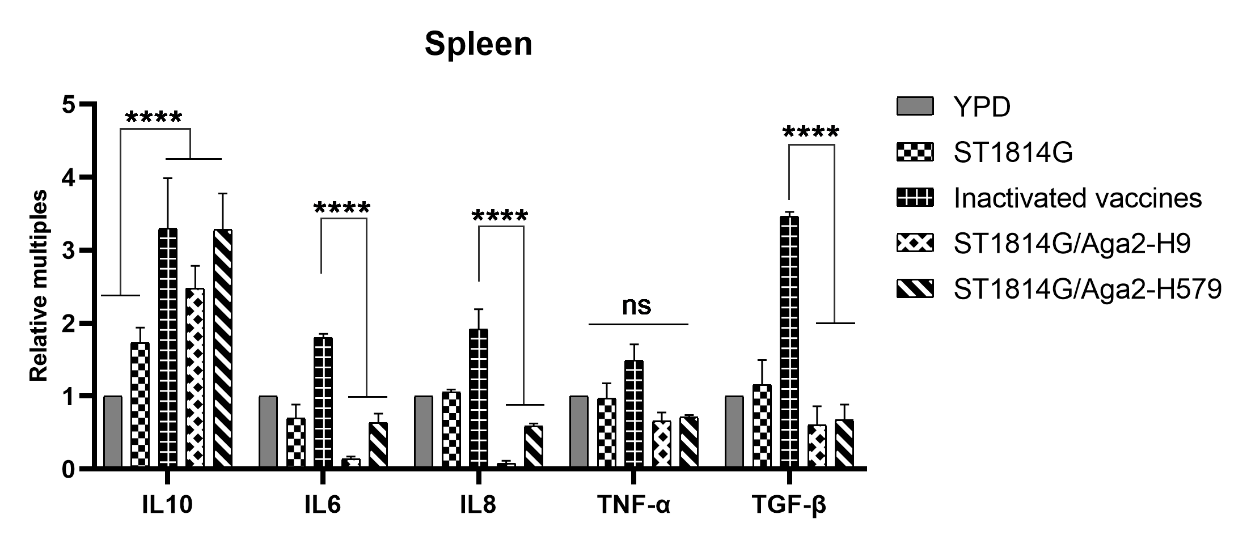

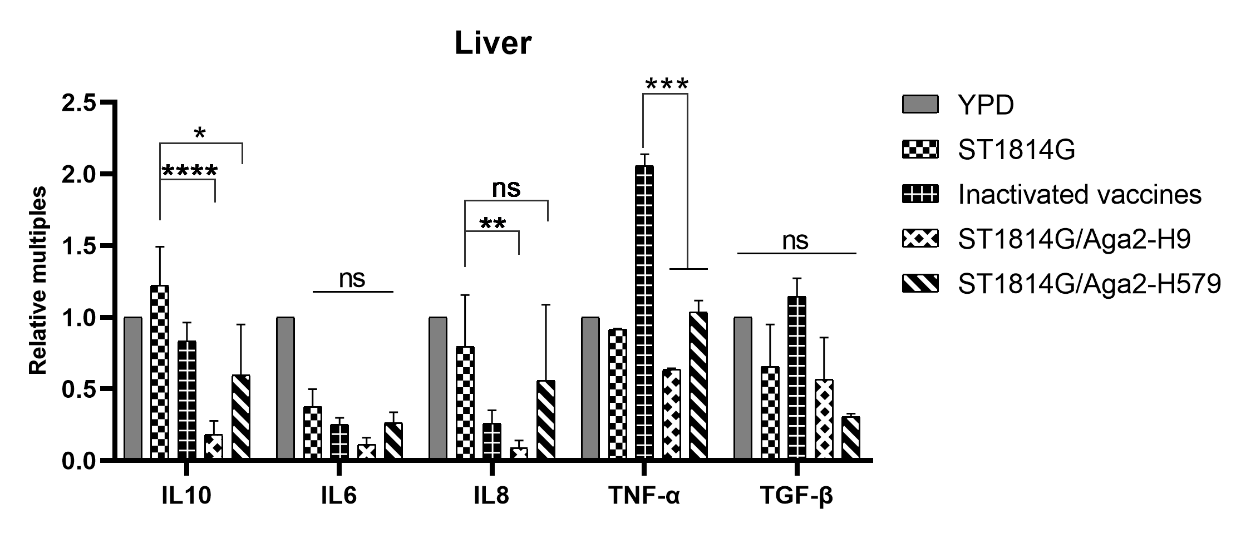


**A**

**B**

**C**

**D**

**Supplementary Figure 3. Effect of oral yeast vaccine on the relative mRNA expression of inflammatory factors and inflammation suppressors.** The mRNA expression of five groups of IL10, IL6 IL8, TNF-α and TGF-β in the Liver(A), Spleen(B) and Bursa of *Fabricius* (BF) (C) and Cecum tonsils (CF) (D). The differences were compared among groups respectively, and the data in figures were obtained from three independent experiments and represent the averages ± SD. The significance of differences was determined by two-way analysis of variance (*, p < 0.05; **, p < 0.01; ***, p < 0.001; ****, p < 0.0001 or ns, no significance).

**Supplementary Table 1. Cloning of important characteristic molecules of ILCs cells in the chicken genome.**

**Supplementary Table1 Primers for PCR amplication**

| **Gene name**  **(chicken）** | **Primer sequence（5'-3'）** | **Size(bp)** | **Ref.** |  |
| --- | --- | --- | --- | --- |
| **CD127** | F:TGCAGCATGCTCAGAATG R:GTATCTGTAACGCCTACTGATT | 1402 | NM_001080106.1 | Successful Cloned |
| **CD117** | F:GCAGTACTCCGCAGCCTC R:TCCAGATGCCACTCAAACATCTT | 2989 | NM_204361.1 | Successful Cloned |
| **ST2** | F:GACTCAGCCCAGTGCAAAG R:GCAGGCATCTTGACAGTAGC | 1104 | AB041737.2 | Successful Cloned |
| **CD25** | F:CAGGTCCCATGGAGCTCAA R:TATCGTCTCTGCTCGGCGA | 664 | NM_204596.1 | Successful Cloned |
| **CD90** | F:TCTGGGCATCTCGTCCTC R:GTGGTTAGCGTTAGATACACG | 656 | NM_204381.2 | Successful Cloned |
| **GATA3** | F:CTCCGATTACAAACCAACCT R:GAGACCGTAAGCATTCAGCA | 1506 | NM_001008444.1 | Successful Cloned |
| **Id2** | F:TTCAGCATGAAAGCTTTCAG R:GTCCGATTCAGCCACAGAG | 418 | NM_205002.2 | Successful Cloned |
| **Id3** | F:TAATTCTCGGCGGTCCCAC R:GGAGGAGGCGTTAGTGAC | 465 | NM_204589.1 | Successful Cloned |
| **RORα** | F:GCCATCTCCAAGCGATCTACAT R:AGTGCTTAGGTGATACGTTTAC | 1625 | NM_001289887.1 | Successful Cloned |
| **IL25** | F:GGCTTTCCCTATTTAGCTGTG R:CAGCCTCAGAAGATGCAAGT | 717 | XM_040682613.2 | Successful Cloned |
| **IL17A** | F:AGATGTCTCCGATCCCTTGTTC R:AGCCTGGTGCTGGATCAGT | 509 | NM_204460.2 | Successful Cloned |
| **IL22** | F:AGCGCTGAGTGCTGTAACT R:GGAGGCATTCCTTCAAGACT | 674 | NM_001199614.1 | Successful Cloned |
| **RUNX3** | F:CTCCTCCTCTTCCTGCAGCT R:GGAGTAACGTCACGGTGG | 1364 | XM_015297733 | Not cloned |
